# Evaluating the Impact of the U.S. National Toxicology Program: A Case Study on Hexavalent Chromium

**DOI:** 10.1289/EHP21

**Published:** 2016-08-02

**Authors:** Yun Xie, Stephanie Holmgren, Danica M. K. Andrews, Mary S. Wolfe

**Affiliations:** 1 Division of the National Toxicology Program, and; 2 Office of the Deputy Director, National Institute of Environmental Health Sciences, National Institutes of Health, Department of Health and Human Services, Research Triangle Park, North Carolina, USA

## Abstract

**Background:**

Evaluating the impact of federally funded research with a broad, methodical, and objective approach is important to ensure that public funds advance the mission of federal agencies.

**Objectives:**

We aimed to develop a methodical approach that would yield a broad assessment of National Toxicology Program’s (NTP’s) effectiveness across multiple sectors and demonstrate the utility of the approach through a case study.

**Methods:**

A conceptual model was developed with defined activities, outputs (products), and outcomes (proximal, intermediate, distal) and applied retrospectively to NTP’s research on hexavalent chromium (CrVI). Proximal outcomes were measured by counting views of and requests for NTP’s products by external stakeholders. Intermediate outcomes were measured by bibliometric analysis. Distal outcomes were assessed through Web and LexisNexis searches for documents related to legislation or regulation changes.

**Results:**

The approach identified awareness of NTP’s work on CrVI by external stakeholders (proximal outcome) and citations of NTP’s research in scientific publications, reports, congressional testimonies, and legal and policy documents (intermediate outcome). NTP’s research was key to the nation’s first-ever drinking water standard for CrVI adopted by California in 2014 (distal outcome). By applying this approach to a case study, the utility and limitations of the approach were identified, including challenges to evaluating the outcomes of a research program.

**Conclusions:**

This study identified a broad and objective approach for assessing NTP’s effectiveness, including methodological needs for more thorough and efficient impact assessments in the future.

**Citation:**

Xie Y, Holmgren S, Andrews DMK, Wolfe MS. 2017. Evaluating the impact of the U.S. National Toxicology Program: a case study on hexavalent chromium. Environ Health Perspect 125:181–188; http://dx.doi.org/10.1289/EHP21

## Introduction

For more than 35 years, the National Toxicology Program (NTP) has conducted research, testing, and analysis activities and has disseminated information about potential health hazards in our environment. As the largest government program in toxicology, NTP has studied more than 2,800 substances for a variety of health effects, developed numerous new methods and tools, and published over 600 reports and monographs (NTP 2014e). NTP staff have also published thousands of peer-reviewed journal articles (NTP 2014e). NTP routinely provides study data, tools, publications, and information about its activities on its public Web site (http://ntp.niehs.nih.gov), along with annual reports to summarize the work of each fiscal year. These efforts communicate NTP’s work to the public and any interested groups (stakeholders). To evaluate the impact of NTP’s work, we sought methods to assess the effectiveness of NTP’s science at advancing toxicology and being translated to public health decision-making.

In 1978, NTP was established within the U.S. Public Health Service in response to growing scientific, regulatory, and congressional concerns that many human diseases and disabilities are linked to chemical exposures (Public Health Service 1978). NTP was created as an interagency program with the goal of improving the coordination and integration of toxicology testing activities on chemicals of public health concern and developing and validating improved testing methods. Thereby, NTP provides needed information to health regulatory and research agencies. Housed administratively at the National Institute of Environmental Health Sciences (NIEHS), National Institutes of Health (NIH), the program focuses on developing and providing scientific information upon which public health decisions are based (Wolfe and Portier 2006).

Evaluating the impact of federally funded research using a broad and methodical approach is necessary to ensure that public funds are advancing the mission of federal agencies. The impact of federally funded research has been evaluated in a number of studies, supported by public universities, private organizations, and federal agencies (Battelle Technology Partnership Practice 2011; Chatterjee and DeVol 2012; Coryn et al. 2007; Ehrlich 2011, 2012; Families USA’s Global Health Initiative 2008; Buxton et al. 2008; Jacob and Lefgren 2011; Liebow et al. 2009; Toole 2007, 2012). In 2013, NIH released a report on approaches for assessing the value of biomedical research (NIH 2013), and in 2014 the National Academy of Sciences published a report related to measuring the impact of research on society (NRC 2014). The literature for assessing the value of research highlights the importance of measuring impact and the challenges involved, such as attribution (finding a connection from research to an impact), lag time (accounting for the potentially long period of time between research and impact), and external factors (research institutions lacking direct control over how their work will be used by others, such as federal agencies, industry, and the public) (Chatterjee and DeVol 2012; Buxton et al. 2008; Liebow et al. 2009; NIH 2013; NRC 2014; Toole 2007, 2012).

Prior to this project, NTP assessed its impact by tracking regulatory actions through *Federal Register* notices for references to its work and through other ad hoc methods. Based on the literature, we aimed to develop a more formal and methodical approach that would yield a broad assessment of NTP’s effectiveness across multiple sectors and demonstrate the utility of the approach using a case study of NTP’s research on hexavalent chromium (CrVI).

Determining the ideal time window to perform an evaluation requires a balance between the availability of records and sufficient lag time to see the full impact of the work. Choosing a project that was completed many decades ago may allow a more thorough search for distal outcomes, such as changes in public health; however, there may be a lack of correspondence and electronic records to provide evidence for proximal and intermediate outcomes. In research fields, there seems to be a rise in mean citation counts for roughly the first 5–10 years after publication (Wang 2013). Thus, to ensure that we would at least capture a good representation of citations from other scientific articles, we considered NTP projects that were completed between 5 and 10 years ago. In addition, to increase the chance of testing all elements in our approach, from proximal to distal outcomes, we wanted to choose a project that began with high profile, external nominations. CrVI was selected for the case study because NTP’s work was completed more than 5 years ago, which is presumably sufficient time to identify its use by stakeholders and evaluate impacts. NTP carried out the research and testing activities on CrVI in contract laboratories and followed established procedures for the analysis, reporting, and peer review of the research findings (NTP 2016b).

## Methods

**Figure 1:**
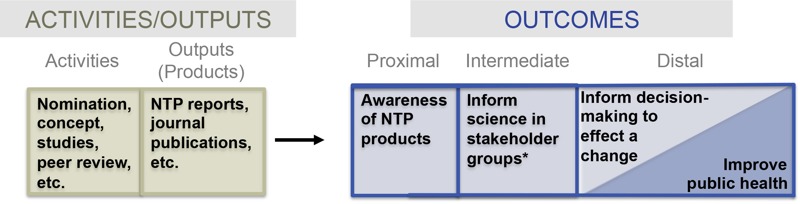
Logic model for evaluating the impact of NTP studies on hexavalent chromium (CrVI). *Stakeholder groups include academia, industry, regulatory, and nonregulatory agencies, state agencies, nongovernment groups, and international groups.

Using a logic model approach (Engel-Cox et al. 2008; W.K. Kellogg Foundation 2004), we defined activities, outputs (products), and outcomes (proximal, intermediate, distal) (Figure 1) for a case study evaluating NTP’s research program on hexavalent chromium (CrVI).

### NTP Activities and Outputs

The logic model accounts first for the main activities in NTP’s research, including the nomination of CrVI to NTP for study, the conduct of studies, and the peer review of NTP study results. These activities led to a number of products, including journal articles and reports. While there were tangential products from NTP studies, such as fact sheets, this assessment focused on NTP’s scientific publications.

The NTP Electronic Library stores research data, documents, and other information for NTP studies. Information about the nomination of CrVI to NTP for study was obtained from the NTP Electronic Library and nomination summary pages (NTP 2014a, 2014b, 2014c), along with the dates for when NTP studies began. The NTP Web site, which maintains an updated list of all NTP reports and journal articles in the peer-reviewed literature, was used to find the NTP reports for CrVI (NTP 2014e). Information about the external peer review of the draft NTP reports was found in the published final reports. NTP journal articles related to chromium were found by consulting NTP staff scientists listed as contributors in the NTP reports for CrVI and by conducting subsequent searches in Web of Science, Scopus, and PubMed for relevant publications using the names of NTP staff scientists listed as contributors in those NTP reports.

### NTP Outcomes: Proximal

Proximal outcomes were associated with stakeholder groups (e.g., academia, industry, nongovernment organizations, federal agencies, state agencies, and international groups) gaining knowledge or awareness of NTP reports and publications. This direct impact was represented by user data from Web-page views of NTP reports and requests for information about NTP’s work. For the CrVI case study, the number of Web-page views for NTP reports was obtained from server logs for July 2011 through October 2014. Web pages with Webtrends code (Webtrends 2016) can track the time of visits to the URL, length of visits, and Internet Protocol (IP) addresses of visitors. External (outside of NIEHS or NIH) versus internal (NIEHS or NIH) visitors can be determined based on the IP addresses and the corresponding companies that own those addresses. The number of public requests from 2005 through 2014 regarding CrVI was obtained from NTP’s Central Data Management, which handles external requests for NTP information or documents. NTP’s Central Data Management follows the program’s correspondence procedures, which allow for a central collection point for incoming correspondence, a coordinated response, and official tracking of NTP’s responses to outside requests for information in oral or written form.

### NTP Outcomes: Intermediate

Intermediate outcomes, or citation metrics of NTP publications (reports and journal articles), were used as a metric of informing science in stakeholder groups. For NTP journal articles (Collins et al. 2010; Levine et al. 2009, 2010; Stout et al. 2009; Witt et al. 2013) citations in other journal articles (reviews, original research articles, and meta analysis) and book chapters were obtained from Web of Science (Thomson Reuters 2016) and Scopus (2016), which are online, subscription-based, scientific citation indexing services, through October 2014. NTP technical reports and toxicity reports are not fully indexed in Web of Science or Scopus. For NTP’s technical report (NTP 2008) and toxicity report (Bucher 2007) on CrVI, PubMed Central (2016), a free full-text archive of biomedical and life sciences journal literature, was used to identify citations in publicly archived articles through 2014. The number of citations was compiled and duplicates were removed. To focus on the use of NTP’s work by external scientists, self-citations by NTP authors were also removed (Aksnes 2003; Glänzel et al. 2006). The citations were categorized by type: contextual/informative and significant. This categorization was conducted by a fact-based analysis of where and how NTP’s work was used in the external paper without taking into account the sentiment of the citation due to difficulties in categorizing the mindset or opinion of external scientists based simply on the text of a scientific article. Identifying the sentiment expressed by documents on a particular topic is challenging (Pang and Lee 2008). Citations categorized as contextual/informative cited NTP’s work to provide context for their study or inform experimental design and data interpretation. For example, NTP’s results may be described in the introduction to provide explanation for why the new work was performed or summarized in the discussion to provide context for the results of the new work. Significant citations used NTP’s data or results in multiple areas of their work or scientific review, such as using NTP’s data in their graphs and tables and comparing their results with NTP’s work in the introduction and discussion.

We used the Query, View, and Report System (QVR), a tool that enables Department of Health and Human Services staff to search, view, and retrieve information from NIH databases about grant applications and awards (NIH 2014), to identify funded grants from January 2000 to July 2014 with chromium in the title or abstract. The reference lists for the selected grants, when available, were manually screened for explicit citations of NTP’s publications on CrVI. This indirect search method for NTP products in the references of grants was necessary because the references and full text of grants are not directly searchable through the QVR tool.

Using Google (2016), citations of NTP’s work in documents from other agencies and groups were searched by keywords: National Toxicology Program or NTP and chromium, hexavalent chromium, chromium 6, or chromium VI. The search was performed in the domains of 50 U.S. states (e.g., ca.gov, wa.gov, ny.gov), U.S. federal agencies (epa.gov, fda.gov, cdc.gov, defense.gov, and osha.gov), U.S. nongovernmental groups (ewg.org, nrdc.org, earthjustice.org, waterrf.org, and sierraclub.org), and the international community (who.int, iarc.fr, inchem.org, hc-sc.gc.ca, and efsa.europa.eu). For each search result, any identified reports and white papers were screened manually for references to NTP’s work on CrVI. Citations of NTP’s work in these types of documents could be explicit, meaning NTP’s work was cited in reference lists or referenced in footnotes, or implicit, meaning NTP’s work was identified through context. For example, in the U.S. Environmental Protection Agency’s (EPA) “Methods to Develop Inhalation Cancer Risk Estimates for Chromium and Nickel Compounds” (U.S. EPA 2011), the “12th Report on Carcinogens” was explicitly referenced; however, the NTP’s technical report on CrVI was implicitly referenced in text: “Further support comes from a recent 2-year chronic bioassay conducted by the National Toxicology Program (NTP) concluding that Cr(VI) is carcinogenic when ingested in drinking water” (U.S. EPA 2011).

Lexis Advance® (LexisNexis 2016), a platform to search legal and government documents, was used to identify congressional testimony and lawsuits that cite NTP’s work. As with the Google searches, the following search terms were used in Lexis Advance: National Toxicology Program or NTP and chromium, hexavalent chromium, chromium 6, or chromium VI. Results were restricted to congressional testimony as well as lawsuits, and the full-text documents were exported. Each full-text document was manually reviewed for mentions of NTP publications.

### NTP Outcomes: Distal

The Google and Lexis Advance® search approaches described above were also applied to identify distal outcomes, or references to NTP’s work that related to changes in regulation(s). All documents were screened manually for explicit or implicit references to NTP’s work on CrVI. Explicit references were found in footnotes or reference lists, while implicit references were found in the text. For example, in California’s “Initial Statement of Reasons” to adopt a maximum contaminant level for CrVI in drinking water, NTP’s work was not present in the reference list (California Department of Public Health 2013). Instead, NTP’s work was described in the text: “In May 2007, National Toxicology Program’s reports on studies on the carcinogenesis of hexavalent chromium (dichromate dihydrate) in drinking water, which found there to be sufficient evidence of carcinogenicity in rodents, were reviewed and approved by the Board of Scientific Counselors Technical Reports Review Subcommittee” (California Department of Public Health 2013).

## Results

### NTP Activities and Outputs

In 2000 and 2001, the California Congressional Delegation, California Environmental Protection Agency, and the California Department of Health Services nominated CrVI to NTP for study (NTP 2014a, 2014b, 2014c). The California nominations were based on concern for the safety of drinking water in several California cities as a result of CrVI contamination. At the time, there was a lack of experimental data on the toxicity of orally ingested CrVI. Hexavalent chromium compounds had already been shown to cause cancer when inhaled from contaminated air, mainly through occupational exposure, and were listed in the “Report on Carcinogens” (NTP 2014d). However, CrVI can be ingested and found in water and soil as a contaminant from various industrial processes including electroplating operations, leather tanning, and textile manufacturing (ATSDR 2012).

**Figure 2:**
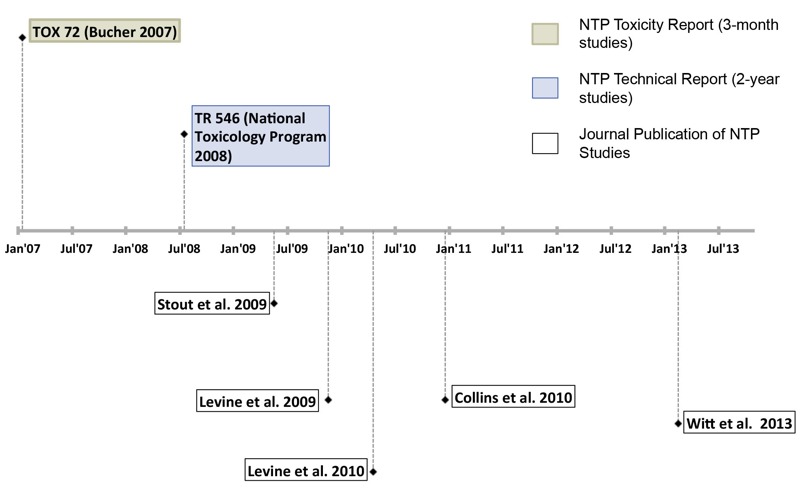
NTP toxicity report (TOX 72, 3-month studies), technical report (TR 546, 2-year studies), and journal articles of NTP studies on sodium dichromate dihydrate, a compound that contains CrVI.

In response to the CrVI nomination, NTP conducted short-term and long-term toxicity and carcinogenicity studies on sodium dichromate dihydrate, a compound that contains hexavalent chromium. NTP’s products on CrVI included one toxicity report (TOX 72) (Bucher 2007), one technical report (TR 546) (NTP 2008), and five journal articles (Figure 2) (Collins et al. 2010; Levine et al. 2009, 2010; Stout et al. 2009; Witt et al. 2013). TOX 72 described NTP’s 3-month studies of CrVI in drinking water in rodents and concluded that sodium dichromate dihydrate caused hyperplasia and ulceration of the stomach in rats, and anemia and lesions of the small intestine in rats and mice. TR 546 described NTP’s 2-year studies of CrVI in drinking water in rodents and concluded that sodium dichromate dihydrate caused oral cancers in rats and cancer of the small intestine in mice. NTP also generated other scientific products related to CrVI that preceded its drinking water studies: *a*) a series of three final reports on the reproductive effects of potassium dichromate administered in the diet in rodents (NTP 1996a, 1996b, 1997) and *b*) the NTP “Report on Carcinogens” listing of CrVI compounds as “known to be human carcinogens” since 1980 (NTP 2014d).

### Proximal Outcomes

Web statistics and external requests showed stakeholders had direct awareness of NTP’s products. From 2005 through 2014, there were 14 requests related to NTP’s work on CrVI (see Table S1). These requests came from different groups including industry, U.S. EPA, U.S. House of Representatives staff, a New Jersey state agency, and academia. NTP did not have the capability to track Web-page views until July 2011. While Web statistics are not available prior to July 2011, the NTP toxicity report TOX 72 and NTP technical report TR 546 received 16,471 and 22,634 views, respectively, from July 2011 through October 2014. About 87% of Web-page views for TR 546 and 77% of Web-page views for TOX 72 were external, meaning the views came from non-NIH and non-NIEHS IP addresses.

### Intermediate Outcomes

**Figure 3:**
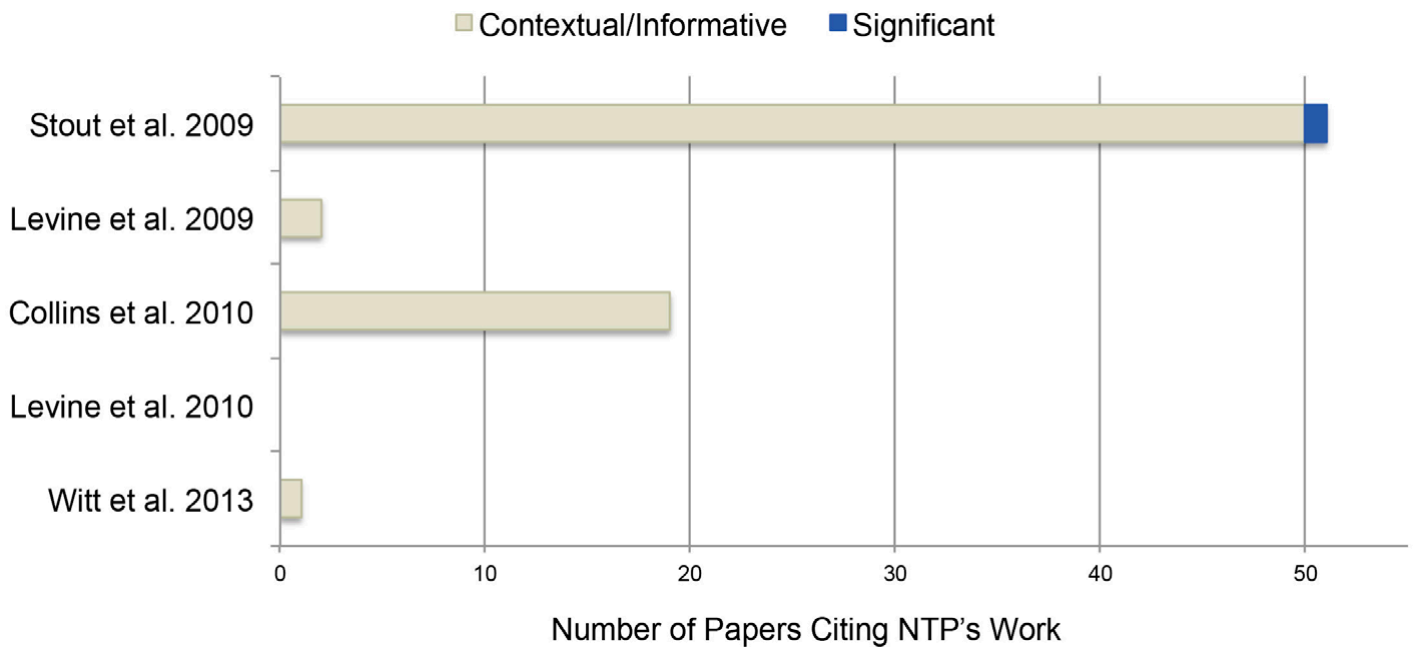
Number and type of citations for NTP journal articles. Contextual/informative citations used NTP products to provide context for their study or to inform experimental design and data interpretation. Significant citations used NTP methods, data, or results as the basis for their work or as comparison with their work.

**Figure 4:**
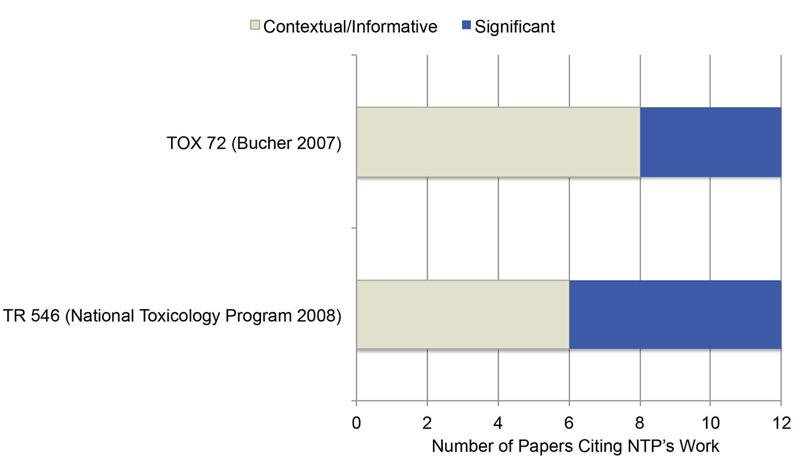
Number and type of citations for NTP technical report (TR 546, 2-year studies) and NTP toxicity report (TOX 72, 3-month studies). Contextual/informative citations used NTP products to provide context for their study or to inform experimental design and data interpretation. Significant citations used NTP methods, data, or results as the basis for their work or as comparison with their work.

With respect to journal articles that cited NTP’s publications, all but one used NTP’s work contextually and informatively (Figure 3), such as using NTP’s work to provide background information in the introduction or discussion. One journal article used an NTP article on CrVI in a significant manner, discussing NTP’s work in the introduction, the background, a table, two figures, and the body of the text (Thompson et al. 2011). While citation information for TR 546 and TOX 72 is limited to publications available in PubMed Central, 8 of the 12 citations found used TOX 72 significantly, and 6 of the 12 citations used TR 546 significantly (Figure 4).

NIH grants were analyzed to determine if NTP products were used as support for future research. Of the roughly 100 NIH funded grants with chromium in the title or abstract (2000–2014), 4 referenced NTP’s work. Two references were for TR 546. The other 2 references were for the “Report on Carcinogens” listing of CrVI compounds.

U.S. states, federal agencies, and nongovernment groups cited NTP’s work to identify CrVI as a hazard (see Tables S2–S4). The World Health Organization’s International Agency for Research on Cancer and International Programme on Chemical Safety also cited NTP’s work with CrVI (see Table S5). Academics, U.S. federal agency leaders, a U.S. senator, and members of nongovernment groups have included references to NTP’s work in congressional hearings (see Figure S1). In addition, NTP has informed the science in three lawsuits (see Table S6).

### Distal Outcomes

**Figure 5:**
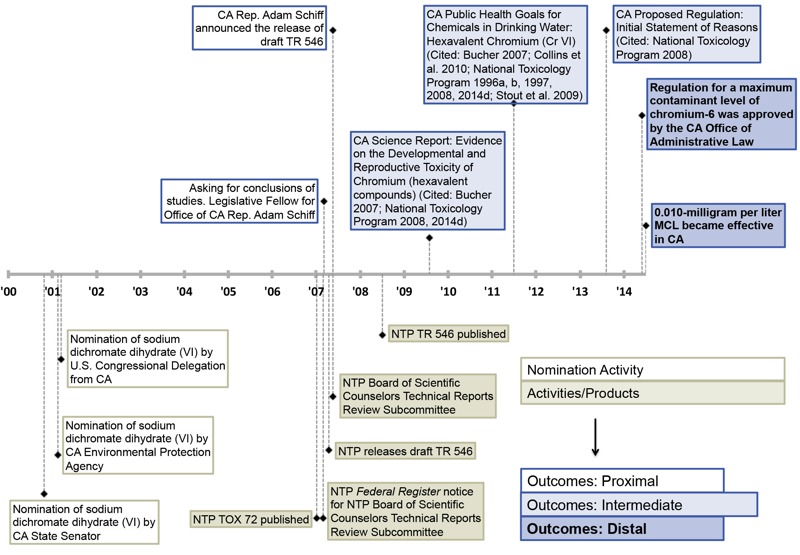
Timeline of activities, outputs, and distal outcomes for NTP’s impact in California (CA).

Of significance, NTP’s work was used to inform decision-making and effect a regulation change in California. NTP’s research was key to the nation’s first-ever drinking water standard for CrVI adopted by California in 2014 (Figure 5). In 2007, when NTP announced in a *Federal Register* notice that draft TR 546 would be peer reviewed and posted publicly, the office of CA Representative Adam Schiff asked for the conclusions of NTP’s toxicology and carcinogenicity studies and announced that NTP had released the draft. Following the release of TR 546 and other NTP publications, California released several reports related to CrVI (Campbell et al. 2009; Chromate Toxicity Review Committee 2001; Office of Environmental Health Hazard Assessment 2000; Pesticide and Environmental Toxicology Branch 2011) and cited a number of NTP’s publications (Figure 5; see also Table S2). This led to a proposed regulation in California that specifically cited TR 546 on the carcinogenicity of CrVI to rodents in drinking water (California Department of Public Health 2013). The California Office of Administrative Law approved the regulation for a maximum contaminant level of CrVI in May 2014. The 0.010-mg/L maximum contaminant level became effective on 1 July 2014 (CalEPA 2016).

NTP’s work on CrVI also had distal impacts at the Department of Defense (DoD) and Occupational Safety and Health Administration (OSHA). In 2011, DoD issued a final rule to amend the Defense Federal Acquisition Regulation Supplement to minimize the use of materials containing CrVI in items they acquire (see Table S7). In 2006, OSHA amended the existing standard that limits occupational exposure to CrVI (see Table S7).

## Discussion

We sought to develop a formal and methodical approach that would yield a broad assessment of NTP’s effectiveness across multiple sectors and demonstrate the utility of the approach through a case study of one of NTP’s research projects. Our approach was to use a logic model and apply it retrospectively to a case study of NTP’s research on CrVI. Because this case study is for assessing NTP’s effectiveness, the logic model focuses on activities, outputs, and outcomes; inputs (e.g., human, financial, and organizational resources) were not evaluated.

We conducted an evaluation of proximal, intermediate, and distal outcomes of NTP’s work in multiple sectors and found challenges in each step. For proximal impacts, we learned that NTP did not track Web-page views for its reports before July 2011. Thus, we are not able to determine the number of downloads for the main products of NTP’s CrVI studies immediately following publication, when interest would presumably be the highest. This also makes it difficult to gauge if the Web-page views for TR 546 and TOX 72 are high or low compared to other NTP projects. For reports published after July 2011, we would be better able to make those kinds of comparisons. For future assessments, it would be important to track Web statistics immediately following the posting of NTP publications online. In addition, it would be worth obtaining information on the number of times NTP articles are downloaded from journal publishers, when available.

For intermediate impact, we learned that NTP’s technical and toxicity reports are not completely indexed in subscription-based, scientific abstract and citation databases, such as Scopus and Web of Science. As a result, in order to identify publications that cite the NTP reports TR 546 and TOX 72, we searched PubMed Central (PMC). PMC is a free archive of full-text, biomedical and life science journal articles that enabled us to search for articles with NTP publications in the references. The number of papers in PMC is limited. There are about 3.7 million articles in PMC (PubMed Central 2016), while Scopus has over 60 million journal records (Elsevier 2016). Because TR 546 and TOX 72 are NTP’s main products for CrVI, they may be cited more frequently in a significant manner than NTP’s journal articles. However, with the current limited dataset from PMC, a complete citation assessment cannot be made. While manual curation could provide additional citation data, such a method would not be thorough. The more important issue is to ensure NTP reports are included in scientific citation indexing services to enable thorough future evaluations.

Assessing whether NTP efforts led to distal outcomes had three main challenges: lag time, external factors, and attribution. First, the lag time between when outputs occur and a specific outcome is implemented can be extensive. With respect to CrVI, the lag time from the release of the draft report on NTP’s 2-year studies of CrVI in drinking water (TR 546) to approval of the maximum contaminant level of CrVI in drinking water by California was 7 years. Any improved public health impact resulting from this regulation may not be known for many years to come.

The second challenge is that the opportunity to effect a change is not always straightforward and may rely on external factors. Whether NTP’s work will lead to drinking water regulation changes in other parts of the United States as in California is beyond NTP’s purview. For example, in Washington State, TR 546 was cited as a supportive scientific development in “Draft Revisions Model Toxics Control Act Method A Groundwater Cleanup Levels” (Toxics Cleanup Program Policy & Technical Support Unit 2010); however, the rulemaking was suspended for a year and then not continued (State of Washington 2010). TR 546 is also referenced in the Senate bill “Protecting Pregnant Women and Children from Hexavalent Chromium Act of 2011” (U.S. Senate 2011). The bill was referred to the Committee on Environment and Public Works, and there has been no action since 2011. Similarly, TR 546 is referenced in the House bill “Protecting Pregnant Women and Children from Hexavalent Chromium Act of 2012” (U.S. Congress 2012). The bill was referred to the Subcommittee on Environment and the Economy, and there has been no action since 2012. These examples illustrate that external factors can influence whether or not NTP products result in distal outcomes.

Finding clear attribution is a third challenge that applies to both intermediate and distal outcomes. While many reports, lawsuits, and other works use NTP publications on CrVI, they often lack consistent or clear citations either in-text or in a reference list. Thus, key word searches for citations to NTP’s work on CrVI often find false positives that must be manually eliminated or may miss true positives. Unlike NIH grants, which have unique identifiers that help NIH track the impact of grants (Boyack and Jordan 2011; Drew et al. 2016), there are no universal citation requirements for many types of work (e.g., lawsuits, government reports, and regulatory actions). Because Web search results were manually evaluated for documents with explicit references to NTP’s work on CrVI or clear in-text descriptions that described NTP’s work and results, we likely underreported NTP’s impacts. For example, we might have missed a reference during manual search or not categorized text descriptions that were unclear in their attribution to NTP’s work.

Finally, for all outcomes, it is difficult to understand the exact nature of NTP’s contribution even when NTP’s work is clearly included in a reference list. Many factors are involved in producing reports, lawsuits, regulations, and other works. It is impossible to assign a level of importance to NTP’s work for any action by an external group; we can only note when NTP had a contribution. When linkages from proximal to distal outcomes can be made, there is a stronger case that NTP’s work made a significant contribution. As was the case for the impact of NTP’s CrVI studies on activities in California, our evaluation methods were able to identify proximal, intermediate, and distal outcomes. In California, there was direct interest and use of NTP’s work in scientific documents, and this led to the reference of NTP’s work in the “Initial Statement of Reasons” (California Department of Public Health 2013) for a maximum contaminant level of CrVI in drinking water.

In carrying out this evaluation, we identified a need for better tools to enhance assessments of a research project’s potential research impacts. While obtaining the number of citations for NTP reports and journal articles in scientific publications is straightforward, performing content analysis to determine how NTP’s work was cited is much more resource intensive. Developing text-mining tools to scan the full text of each citation would allow quicker content analysis to be performed. Better methods are also needed to search for references in grants to determine if NTP’s work was used to support the proposed new projects. Currently, the references of NIH grants are not searchable through the QVR tool. Indirect searches have to be performed, such as searching for grants that relate to chromium and then searching relevant grant files for a list of references. This indirect search method is likely to yield an incomplete list of citations. Finally, there is a need for Web-mining tools to increase the efficiency and completeness of searches for explicit and implicit citations of NTP’s work in documents from state agencies, federal agencies, nongovernment groups, and international groups. Searching through the Web for outcomes was a time-consuming manual process that highlighted the need for automation. With Web- and text-mining software, NTP may more thoroughly and efficiently identify potential impacts. If such tools can be developed, impact searches could be performed on a regular basis and stored in a database for public access to enhance the communication of NTP’s work.

The purpose of evaluating NTP’s impact is 3-fold: (1) collect and analyze data to learn how NTP is achieving its goals; (2) implement improvements based on what is learned; (3) following implementation of these improvements, use the methods developed here to evaluate whether the changes have helped NTP achieve its goals. For example, this case study identified that TR 546 was used more often than NTP journal articles in documents by nongovernment organizations, federal agencies, state agencies, and international groups (see Tables S2–S7 and Figure S1). There are limited data for citations of TR 546 in scientific literature due to inconsistent indexing in subscription-based, scientific abstract and citation databases. However, the available data suggest that TR 546 has more significant citations in scientific literature than NTP journal articles. NTP technical reports, like TR 546, are more comprehensive with regard to study data and results than to journal articles. NTP journal articles usually focus on a deeper discussion of a subset of the data and results than are available in corresponding technical reports. Thus, it is possible that stakeholders have greater interest in NTP technical reports because they are a more complete reporting of data and results. It would benefit the goal of NTP to increase the discoverability of its data and results and to harmonize the format of this information for improved usability in databases like the NTP Chemical Effects in Biological Systems (CEBS) database (NTP 2016a). While all NTP reports are publicly available on the NTP Web site (NTP 2014e), by improving discoverability and harmonization, NTP’s data and results should become easier to find and use by big data efforts such as read-across activities (Hartung 2016; Luechtefeld et al. 2016). The methods in this case study could then be adapted to track the use of NTP data and results from databases like CEBS in the future.

## Conclusions

We developed an approach with methodical steps to evaluate a research project’s proximal, intermediate, and distal outcomes. The approach we developed can be applied to future projects for assessing NTP’s effectiveness. The logic model is adaptable to different NTP research programs and could cover outcomes not measured in this case study if relevant to the research topic. By applying this approach to a case study, data and methodological gaps were highlighted. As a result, NTP is seeking to improve indexing of its reports in online scientific citation and abstract databases. NTP is also working to submit full-text reports to PubMed Bookshelf (National Center for Biotechnology Information 2016), which provides free online access to books and documents, as an additional avenue for the public and interested groups to access study results. In addition, NTP aims to improve the discoverability and usability of study data and results within NTP’s CEBS database. While this case study was performed manually, the developed methods and lessons learned could be translated to text- and Web-mining software for more thorough and efficient impact assessments. NTP is developing new text- and Web-mining tools to gather data for impact evaluations on numerous projects on a regular basis. This larger dataset will enable further analysis to identify opportunities for improvement in achieving NTP’s goals. Going forward, we plan to use the approach described in this case study to assess a much larger pool of chemicals studied by NTP.

## Supplemental Material

(940 KB) PDF
