# Supplementary material for: Evaluating the Impact of the U.S. National Toxicology Program: A Case Study on Hexavalent Chromium
Source: Environ Health Perspect. 2016 Aug 2;125(2):181–8. doi: 10.1289/EHP21 (PMC5289905; doi:10.1289/EHP21)
Supplement: (940 KB) PDF [file EHP21.s001.acco.pdf]

Environ Health Perspect
DOI: 10.1289/EHP21

Note to readers with disabilities: *EHP* strives to ensure that all journal content is accessible to all readers. However, some figures and Supplemental Material published in *EHP* articles may not conform to 508 standards due to the complexity of the information being presented. If you need assistance accessing journal content, please contact ehp508@niehs.nih.gov. Our staff will work with you to assess and meet your accessibility needs within 3 working days.

Supplemental Material

Evaluating the Impact of the U.S. National Toxicology Program: A Case Study on Hexavalent Chromium

Yun Xie, Stephanie Holmgren, Danica M. K. Andrews, and Mary S. Wolfe

Table of Contents

Table S1. Requests for NTP's Work 2

Table S2. Intermediate Outcomes in U.S. States 3

Table S3. Intermediate Outcomes in U.S. Federal Agencies 6

Table S4. Intermediate Outcomes in Nongovernment Groups 8

Table S5. Intermediate Outcomes in International Groups 9

Table S6. Intermediate Outcomes in Lawsuits 10

Table S7. Distal Outcomes Found in *Federal Register* Notices 11

Figure S1. Intermediate Outcomes in U.S. Congressional Hearings 12

Table S1. Requests for NTP's Work Recorded by NTP's Central Data Management, 2005-2014

Year of Request	Requestor Group	Information Requested
2006	State agency	Abstract for TOX 72 (Bucher 2007)
2006	Industry	Data from draft TR 546 (National Toxicology Program 2008)
2006	Federal agency	Status of studies for draft TR 546 (National Toxicology Program 2008)
2007	Federal agency	Access to TOX 72 (Bucher 2007)
2007	Unknown	Hardcopy of TOX 72 (Bucher 2007)
2007	State agency	Draft TR 546 (National Toxicology Program 2008) that will be discussed at the December 6, 2007 NTP Board of Scientific Counselors meeting
2007	Industry	Data and results from draft TR 546 (National Toxicology Program 2008)
2007	Industry	Public comments from the review of draft TR 546 (National Toxicology Program 2008)
2007	Congress	Study results from draft TR 546 (National Toxicology Program 2008)
2008	Nongovernment group	Final version of TR 546 (National Toxicology Program 2008)
2009	Industry	Hardcopy of TR 546 (National Toxicology Program 2008)
2010	Industry	Data from TR 546 (National Toxicology Program 2008)
2011	Industry	Listing information from the RoC (National Toxicology Program 2014*) for hexavalent chromium
2011	Academia	Data from TR 546 (National Toxicology Program 2008)

*Note, the Report on Carcinogens is a cumulative report that includes 243 listings since 1980, and the 13th Report (National Toxicology Program 2014) replaces previous editions.

Table S2. Intermediate Outcomes of NTP's Work on CrVI in U.S. States

**U.S. State**	**Year**	**Title** (accessed June 2016)	**NTP Product Cited**
California	2000	Determination of Noncancer Chronic Reference Exposure Levels Batch 2A. Chronic Toxicity Summary: Hexavalent Chromium (Soluble Compounds) [http://oehha.ca.gov/media/downloads/crnr/acrol-cresol.pdf]	National Toxicology Program 1996a, b, 1997
	2001	Scientific Review of Toxicological and Human Health Issues Related to the Development of a Public Health Goal for Chromium(VI): Report Prepared by the Chromate Toxicity Review Committee [http://oehha.ca.gov/media/downloads/public-information/press-release-water/crpanelrptfinal901.pdf]	National Toxicology Program 1996a, b, 1997
	2009	Evidence on the Developmental and Reproductive Toxicity of Chromium (Hexavalent Compounds) [http://oehha.ca.gov/media/downloads/proposition-65/chemicals/chrome0908.pdf]	Bucher 2007; National Toxicology Program 2008, 2014*
	2011	Public Health Goals for Chemicals in Drinking Water Hexavalent Chromium (Cr VI) [http://oehha.ca.gov/media/downloads/water/public-health-goal/cr6phg072911.pdf]	National Toxicology Program 1996a, b, 1997; Bucher 2007; National Toxicology Program 2008; Stout et al. 2009; Collins et al. 2010; National Toxicology Program 2014*
	2013	Hexavalent Chromium MCL: Initial Statement of Reasons [http://www.cdph.ca.gov/services/DPOPP/regs/Documents/DPH-11-005HCMCLISOR.pdf]	National Toxicology Program 2008[a]

Missouri	2010	Pre-Cerclis Site Screening Report: Tannery Sludge Farm Fields Site Andrew, Buchanan, Clinton, and DeKalb Counties, Missouri [http://www.dnr.mo.gov/env/hwp/docs/1011tanneryfieldrpt.pdf]	National Toxicology Program 2008
New Jersey	2008	Health Consultation: Analysis of Lung Cancer Incidence Near Chromium-Contaminated Sites In New Jersey (A/K/A Hudson County Chromium Sites)<br>Jersey City, Hudson County, New Jersey [http://www.state.nj.us/dep/dsr/chromium/chromium-contaminated sites-nj-093008.pdf]	National Toxicology Program 2008, 2014*
	2009	Derivation of Ingestion-Based Soil Remediation Criterion for Cr^{+6} Based on the NTP Chronic Bioassay Data for Sodium Dichromate Dihydrate [http://www.state.nj.us/dep/dsr/chromium/soil-cleanup-derivation.pdf]	Bucher 2007; National Toxicology Program 2008
	2010	Health Consultation: E.C. Electroplating (a/k/a Garfield Chromium Groundwater Contamination Site) Garfield, Bergen County, New Jersey [http://www.state.nj.us/health/eohs/bergen/garfield/ec_electroplating/garfield health_consultation4_10.pdf]	National Toxicology Program 2008
	2010	Health Consultation:<br>Analysis Of Oral, Esophageal and Stomach Cancer Incidence Near Chromium-Contaminated Sites in Jersey City (a/k/a Hudson County Chromium Sites)<br>Jersey City, Hudson County, New Jersey [http://www.state.nj.us/health/eoh/cehsweb/documents/hudson_co_chromium_hc.pdf]	National Toxicology Program 2008, 2014*
New York	2014	Public Comment Draft<br>Public Health Assessment: Gowanus Canal<br>City of New York Borough of Brooklyn<br>Kings County, New York<br>[https://www.health.ny.gov/environmental/investigations/gowanus/docs/pha_comment.pdf]	National Toxicology Program 2008, 2014*
Texas	2013	Public Health Assessment: Van der Horst USA Corporation Terrell, Kaufman County, Texas [http://www.atsdr.cdc.gov/HAC/pha/VanderHorstUSACorp/VanderHorstUSACorpPHAFinal07182013	National Toxicology Program 2008[b]

		_508.pdf]	
	2014	Development Support Document: Hexavalent Chromium (Particulate Compounds) [http://www.tceq.texas.gov/assets/public/implementation/tox/dsd/final/august2014/hexavalent_chromium.pdf]	National Toxicology Program 2014*
Washington	2010	Draft Revisions MTCA Method A Groundwater Cleanup Levels [http://www.ecy.wa.gov/programs/tcp/regs/2009MTCA/AdvGrpMeetingInfo/mtg_100621/MTCA_CleanupLevelRevisionsDiscussionMaterialJun_21_2010.pdf]	National Toxicology Program 2008

[a]Descriptive reference in text.

[b]Implicit citation through reference of press release that announces the results from draft TR 546.

*Note, the Report on Carcinogens is a cumulative report that includes 243 listings since 1980, and the 13th Report (National Toxicology Program 2014) replaces previous editions.

Table S3. Intermediate Outcomes of NTP's Work on CrVI in U.S. Federal Agencies

**U.S. Federal Agency**	**Year**	**Title** (accessed June 2016)	**NTP Product Cited**
Agency for Toxic Substances and Disease Registry, Centers for Disease Control and Prevention (CDC)	2010	Public Health Advisory: E.C. Electroplating (a/k/a Garfield Chromium Groundwater Contamination Site) Garfield, Bergen County, New Jersey [http://www.atsdr.cdc.gov/HAC/pha/ECElectroplating09282010/PublicHealthAdvisoryECElectroplating9292010.pdf]	National Toxicology Program 2008
	2012	Toxicological Profile for Chromium [http://www.atsdr.cdc.gov/toxprofiles/tp7.pdf]	National Toxicology Program 1996a, b, 1997; Bucher 2007; National Toxicology Program 2008, 2014*
National Institute for Occupational Safety and Health, CDC	2013	Criteria for a Recommended Standard: Occupational Exposure to Hexavalent Chromium [http://www.nasf.org/pdfs/regulation/niosh-occupational-exposure-chromium.pdf]	National Toxicology Program 1996a, b, 1997, 2008; Stout et al. 2009; National Toxicology Program 2014*
Environmental Protection Agency	1998	Toxicological Review of Hexavalent Chromium in Support of Summary Information on the Integrated Risk Information System (IRIS) [https://cfpub.epa.gov/ncea/iris/iris_documents/documents/toxreviews/0028tr.pdf]	National Toxicology Program 1996a, b, 1997
	2008	Cancer Assessment Document: Evaluation of the Carcinogenic Potential of Inorganic Hexavalent Chromium (Cr(VI)) [https://www.regulations.gov/document?D=EPA-HQ-OPP-2003-0250-0089]	Bucher 2007; National Toxicology Program 2008
	2010	Draft Toxicological Review of Hexavalent Chromium in Support of Summary Information on the Integrated Risk Information System (IRIS)	National Toxicology Program 1996a,

		[http://ofmpub.epa.gov/eims/eimscomm.getfile?p_download_id=498828]	b, 1997; Bucher 2007; National Toxicology Program 2008; Stout et al. 2009
	2011	Methods to Develop Inhalation Cancer Risk Estimates for Chromium and Nickel Compounds [http://www.epa.gov/ttn/atw/utility/ni_cr_methods_final_report_cover.pdf]	National Toxicology Program 2008,[a] 2014*
	2013	Scientific Workshop: Factors Affecting the Reduction and Absorption of Hexavalent Chromium in the Gastrointestinal (GI) Tract: Potential Impact on Evaluating the Carcinogenicity of Ingested Hexavalent Chromium [https://ofmpub.epa.gov/eims/eimscomm.getfile?p_download_id=524018]	National Toxicology Program 2008; Stout et al. 2009; Collins et al. 2010; Witt et al. 2013
	2014	Preliminary Materials for the Integrated Risk Information System (IRIS) Toxicological Review of Hexavalent Chromium Part 1: Experimental Animal Studies [https://ofmpub.epa.gov/eims/eimscomm.getfile?p_download_id=524795]	National Toxicology Program 1996a, b, 1997; Bucher 2007; National Toxicology Program 2008; Stout et al. 2009; National Toxicology Program 2014*
	2014	Preliminary Materials for the Integrated Risk Information System (IRIS) Toxicological Review of Hexavalent Chromium Part 2: Human, Toxicokinetic, and Mechanistic Studies [https://ofmpub.epa.gov/eims/eimscomm.getfile?p_download_id=524857]	Collins et al. 2010

[a]Descriptive reference in text.

*Note, the Report on Carcinogens is a cumulative report that includes 243 listings since 1980, and the 13th Report (National Toxicology Program 2014) replaces previous editions.

Table S4. Intermediate Outcomes of NTP's Work on CrVI in Nongovernment Groups

**Group**	**Year**	**Title** (accessed June 2016)	**NTP Product Cited**
Earth Justice	2011	EPA's Blind Spot: Hexavalent Chromium in Coal Ash. Coal ash may be the secret source of cancer-causing chromium in your drinking water [http://earthjustice.org/sites/default/files/CoalAshChromeReport.pdf]	National Toxicology Program 2008[a]
Environmental Working Group	2010	Chromium-6 in U.S. Tap Water [http://static.ewg.org/reports/2010/chrome6/chrome6_report_2.pdf]	National Toxicology Program 2008
Water Research Foundation	2012	State of the Science of Hexavalent Chromium in Drinking Water [http://www.waterrf.org/resources/Lists/PublicProjectPapers/Attachments/2/4404_ProjectPaper.pdf]	National Toxicology Program 2008; Stout et al. 2009; Collins et al. 2010
Natural Resources Defense Council	2010	Congress Must Protect People from Toxic Chemicals Known to Cause Harm: Hexavalent Chromium [https://www.nrdc.org/health/files/hexavalentChromium.pdf]	National Toxicology Program 2008

[a]Descriptive reference in text and implicit citation through reference of fact sheet that describes results from TR546.

Table S5. Intermediate Outcomes of NTP's Work on CrVI in International Groups

**Group**	**Year**	**Title** (accessed June 2016)	**NTP Product Cited**
International Agency for Research on Cancer, World Health Organization (WHO)	2012	IARC Monographs - 100C: Chromium (VI) Compounds. [http://monographs.iarc.fr/ENG/Monographs/vol100C/mono100C-9.pdf]	National Toxicology Program 2008, 2014*
International Programme on Chemical Safety, WHO	2013	Concise International Chemical Assessment Document 78: Inorganic Chromium(VI) Compounds [http://www.inchem.org/documents/cicads/cicads/cicad_78.pdf]	National Toxicology Program 1996a, b, 1997; Bucher 2007; National Toxicology Program 2008

*Note, the Report on Carcinogens is a cumulative report that includes 243 listings since 1980, and the 13th Report (National Toxicology Program 2014) replaces previous editions.

Table S6. Intermediate Outcomes of NTP's Work on CrVI in Lawsuits

**Year**	**Lawsuit** (accessed June 2016)	**NTP Product Cited**
2002	Public Citizen Health Research Group; the Paper, Allied-Industrial, Chemical & Energy Workers International Union, v. Elaine CHAO, Secretary of Labor; Occupational Safety and Health Administration (United States Court of Appeals, Third Circuit) [http://caselaw.findlaw.com/us-3rd-circuit/1262699.html]	National Toxicology Program 2014[a]*
2010	Interfaith Community Organization, Inc., Graco Community Org., and Natural Resources Defense Council, Inc. v. PPG Industries, Inc. (United States District Court for the District of New Jersey) [http://cases.justia.com/federal/district-courts/new-jersey/njdce/2:2009cv00480/224458/41/0.pdf?ts=1411556856]	National Toxicology Program 2008[a]
2012	Natural Resources Defense Council, Inc., and Environmental Working Group v. California Department of Public Health; and Ron Chapman, Director of the California Department of Public Health and State Public Health Officer, in his official capacity (Superior Court for the State of California for the County of Alameda) [http://www.cnsenvironmentallaw.com/2012/08/16/NRDC.pdf]	National Toxicology Program 2014*

[a]Descriptive reference in text.

*Note, the Report on Carcinogens is a cumulative report that includes 243 listings since 1980, and the 13th Report (National Toxicology Program 2014) replaces previous editions.

Table S7. Distal Outcomes Found in *Federal Register* Notices

**U.S. Federal Agency**	**Year**	**Federal Register Title** (accessed June 2016)	**NTP Product Cited**
Department of Defense	2011	Final Rule: Defense Federal Acquisition Regulation Supplement; Minimizing the Use of Materials Containing Hexavalent Chromium (DFARS Case 2009–D004)[a] [http://www.gpo.gov/fdsys/pkg/FR-2011-05-05/html/2011-10882.htm]	National Toxicology Program 2014*
Occupational Safety and Health Administration	2006	Final Rule: Occupational Exposure to Hexavalent Chromium[b] [https://www.osha.gov/pls/oshaweb/owadisp.show_document?p_id=18599&p_table=federal_register]	National Toxicology Program 1996a, b, 1997,[c] 2014*

[a] The Department of Defense issued a final rule to amend the Defense Federal Acquisition Regulation Supplement to minimize the use of materials containing CrVI in items they acquire.

[b] The Occupational Safety and Health Administration amended the existing standard that limits occupational exposure to CrVI.

[c]Descriptive reference in text.

*Note, the Report on Carcinogens is a cumulative report that includes 243 listings since 1980, and the 13th Report (National Toxicology Program 2014) replaces previous editions.

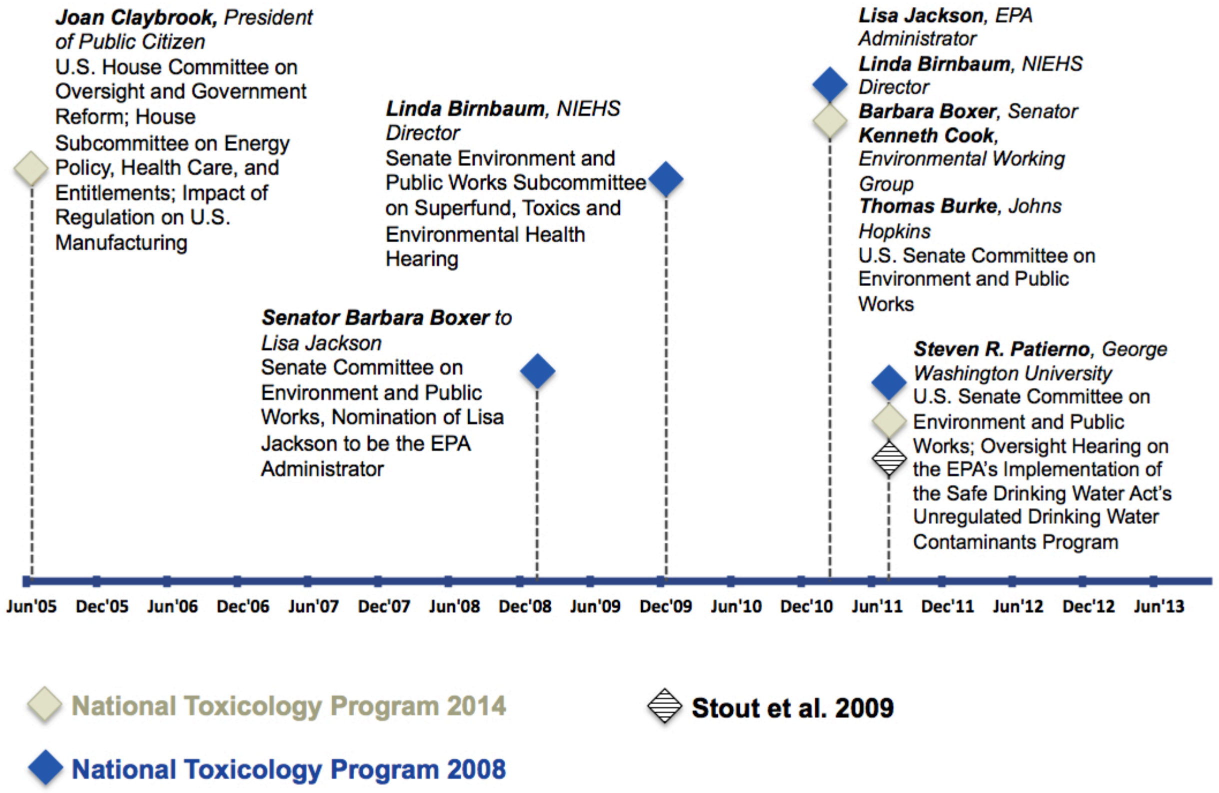

Figure S1. Intermediate Outcomes of NTP's Work on CrVI Mentioned in U.S. Congressional Hearings (Claybrook 2005; Birnbaum 2009; Boxer 2009; Jackson et al. 2011; Patierno 2011). Note, the Report on Carcinogens is a cumulative report that includes 243 listings since 1980, and the 13th Report (National Toxicology Program 2014) replaces previous editions.

References

Birnbaum L. 2009. Testimony Before the Subcommittee on Superfund, Toxics and Environmental Health Committee on Environment and Public Works United States Senate. Washington, DC. Available: http://www.epw.senate.gov/public/_cache/files/fd526d6b-2765-4e09-bf41-631458fd083b/senateepwbirnbaumtscareformfinal.pdf [accessed 22 June 2016].

Boxer B. 2009. Hearing on the Nominations of Lisa P. Jackson to Be Administrator of the U.S. Environmental Protection Agency and Nancy Helen Sutley to Be Chairman of the Council on Environmental Quality. Hearing Before the Committee on Environment and Public Works. United States Senate. One Hundred Eleventh Congress. First Session. Washington, DC. Available: https://www.gpo.gov/fdsys/pkg/CHRG-111shrg94020/pdf/CHRG-111shrg94020.pdf [accessed 22 June 2016].

Bucher JR. 2007. NTP Technical Report on the Toxicity Studies of Sodium Dichromate Dihydrate (CAS No. 7789-12-0) Administered in Drinking Water to Male and Female F344/N Rats and B6C3F 1 Mice and Male BALB/c and am3-C57BL/6 Mice. NTP Toxicity Report 72. Research Triangle Park, NC. Available: http://ntp.niehs.nih.gov/ntp/htdocs/ST_rpts/tox072.pdf [accessed 23 October 2014].

Claybrook J. 2005. The Impact of Regulation on U.S. Manufacturing: Spotlight on Department of Labor and Department of Transportation. Hearing before the Subcommittee on Regulatory Affairs of the Committee on Government Reform. House of Representatives. One Hundred Ninth Congress. First Session. Washington, DC. Available: https://www.gpo.gov/fdsys/pkg/CHRG-109hhrg23627/pdf/CHRG-109hhrg23627.pdf [accessed 22 June 2016].

Collins BJ, Stout MD, Levine KE, Kissling GE, Melnick RL, Fennell TR, et al. 2010. Exposure to hexavalent chromium resulted in significantly higher tissue chromium burden compared with trivalent chromium following similar oral doses to male F344/N rats and female B6C3F1 mice. Toxicol Sci 118:368-379; DOI: 10.1093/toxsci/kfq263.

Jackson L, Birnbaum L, Boxer B, Cook K, Burke T. 2011. Public Health and Drinking Water Issues. Hearing Before the Committee on Environment and Public Works. United States Senate. One Hundred Twelfth Congress. First Session. Washington, DC. Available: https://www.gpo.gov/fdsys/pkg/CHRG-112shrg85223/pdf/CHRG-112shrg85223.pdf [accessed 22 June 2016].

National Toxicology Program. 1996a. Final Report on the Reproductive Toxicity of Potassium Dichromate (Hexavalent) (CAS No. 7778- 50-9) Administered in Diet to SD Rats. Research Triangle Park, NC.

National Toxicology Program. 1996b. Final Report on the Reproductive Toxicity of Potassium Dichromate (Hexavalent) (CAS No. 7778- 50-9) Administered in Diet to BALB/c Mice. Research Triangle Park, NC.

National Toxicology Program. 1997. Final Report on the Reproductive Toxicity of Potassium Dichromate (CAS No. 7778-50-9) Administered in Diet to BALB/c Mice. Research Triangle Park, NC.

National Toxicology Program. 2008. Toxicology and Carcinogenesis Studies of Sodium Dichromate Dihydrate (CAS No. 7789-12-0) in F344/N Rats and B6C3F1 Mice (Drinking Water Studies). NTP Technical Report 546. Research Triangle Park, NC. Available: http://ntp.niehs.nih.gov/ntp/htdocs/lt_rpts/tr546.pdf [accessed 22 June 2016].

National Toxicology Program. 2014. Report on Carcinogens, Thirteenth Edition. Research Triangle Park, NC. Available: http://ntp.niehs.nih.gov/go/roc [accessed 16 June 2016].

Patierno SR. 2011. Oversight Hearing on the Environmental Protection Agency's Implementation of the Safe Drinking Water Act's Unregulated Drinking Water Contaminants Program. U.S. Senate Subcommittee on Environment and Public Works. Washington, DC. Available: http://www.epw.senate.gov/public/_cache/files/09c4210a-f6ef-4632-bd11-377fcd07249e/patiernosenatetestimony71011final.pdf [accessed 22 June 2016].

Stout MD, Herbert RA, Kissling GE, Collins BJ, Travlos GS, Witt KL, et al. 2009. Hexavalent chromium is carcinogenic to F344/N rats and B6C3F1 mice after chronic oral exposure. Environ Health Perspect 117:716-722; DOI: 10.1289/ehp.0800208.

Witt KL, Stout MD, Herbert RA, Travlos GS, Kissling GE, Collins BJ, et al. 2013. Mechanistic insights from the NTP studies of chromium. Toxicol Pathol 41:326-342; DOI: 10.1177/0192623312469856.
